# The feasibility and safety of repeat cryosurgical ablation of localized prostate cancer

**DOI:** 10.1186/s12957-015-0753-9

**Published:** 2015-12-21

**Authors:** Mahmoud Mustafa, Scott Delacroix, John F. Ward, Louis Pisters

**Affiliations:** Urology Department, Faculty of Medicine and Health Science, An-Najah National University, An-Najah University Hospital, Nablus, West bank Palestine; Urology Department, MD Anderson Cancer Center, University of Texas, Houston, TX USA

**Keywords:** Cryoablation, Prostate, Prostate cancer, Repeat cryoablation

## Abstract

**Background:**

The aim of the study was to assess the morbidity and efficacy of repeat cryoablation (CA) in the treatment of localized prostate cancer.

**Methods:**

Twenty-seven patients with median age of 71 years (range 48–80) who underwent repeat CA between April 2003 and April 2011 at a single institution were included. The median initial prostate-specific antigens (PSA) and Gleason values were 6.2 ng/ml (range 4–23.6) and 7 (range 6–9), respectively. Twenty-four patients underwent two CA treatments, and three patients underwent three CA treatments. Pre- and perioperative parameters and oncological and functional outcomes were evaluated.

**Results:**

No intraoperative complications occurred. After the first CA, PSA was undetectable in 10 patients, and the median nadir PSA value was 0.65 ng/ml (range 0.1–4.9). After the second CA, 4 patients had undetectable PSA, and the median nadir PSA value was 1.25 ng/ml (range 0.2–7.9). For patients who underwent a third CA treatment, no patients had undetectable PSA, and the subsequent median nadir PSA value was 1.6 ng/ml (range 0.4–4.5). Two patients had incontinence (1 pad per day) following repeat CA. One patient had urinary retention after the third CA treatment, and one had urethral stricture*.* The mean hospitalization and follow-up periods were 1 day (range 0–2) and 51.5 months (range 11–96), respectively.

**Conclusions:**

Repeat CA successfully reduced PSA levels, and complications were modest. We conclude that repeat CA is a feasible, safe, and effective treatment option for localized prostate cancer.

## Background

With the introduction of prostate-specific antigen (PSA) screening, an increasing number of men are being diagnosed with low-grade, low-stage, and small-volume cancers that are potentially biologically indolent. Choosing whether and how to treat such tumors remains challenging. Men newly diagnosed with low-risk prostate cancer are frequently treated with standard therapies, i.e., radical prostatectomy (RP), external beam radiation therapy, brachytherapy, androgen deprivation therapy, or conservative management [1]. These therapies are associated with high overall cancer-specific and biochemical recurrence-free survival; however, RP, radiation therapy, and androgen deprivation therapy are accompanied by side effects that may negatively affect patients’ health-related quality of life. Conservative management was reported to induce anxiety and elevate stress: however, active surveillance for low-risk prostate cancer was strongly supported by Klotz et al. in many recent studies [2, 3]. As such, renewed interest has emerged in using minimally invasive approaches such as CA to treat clinically localized prostate cancer.

Cryotherapy has become more widespread in practice because of a better understanding of cryobiology [4], introduction of third-generation cryoprobes, and improvement in biopsy and imaging techniques that have enhanced the ability to map the foci and locations of tumors within the prostate, subsequently reducing morbidity while improving effectiveness [4–6]. Information is lacking on whether CA is effective in the repeat setting when disease recurs after initial CA, and there is no formal definition of CA eligible tumor. Moreover, morbidity associated with CA has been primarily reported from single hospital-based studies, typically in highly selected patients [7–13]. Herein, we are the first to report on the morbidity and efficacy of repeat CA as a primary and salvage option for the treatment of clinically localized prostate cancer.

## Methods

We retrospectively evaluated the hospital records of patients who underwent a repeat CA procedure at a single institution between April 2003 and April 2011. Twenty-seven patients with median age 71 years (range 48–80) underwent repeat CA for biopsy-proven prostate cancer. Repeat cryotherapy procedures were performed with similar methodology to the initial salvage cryotherapy procedure which has been described in detail elsewhere. [14]. All procedures were performed under general anesthesia with the urethral warming catheter in place and set at a temperature of 42 °C to protect the urethra. Whole-gland CA was applied except in five patients where focal therapy was used. The median PSA value and Gleason score at initial diagnosis were 6.2 ng/ml (range 4–23.6) and 7 (range 6–9), respectively. The clinical stages were T1c in 15 patients, T2 in 11 patients, and T3 in 1 patient. The preoperative demographic characteristics and functional and oncological outcomes were summarized in Table 1. All patients had negative bone scan and computed tomography findings prior to application of CA. Staging transrectal ultrasonography was performed to rule out seminal vesicle involvement. Biopsy of the seminal vesicles is obtained at the time of transrectal ultrasound-guided prostate biopsies. After the prostate biopsies are obtained, the seminal vesicle tissue is visualized ultrasonographically, and two cores are obtained of each seminal vesicle. Fifteen patients (56 %) had undergone CA as primary therapy; however, 12 patients (44 %) had had hormone therapy (HT) and/or radiation therapy prior to first CA (Table 1). Neoadjuvant HT in 10 patients over a median period of 7 months (range 3–96). Twenty-four patients underwent two cryosurgical ablation procedures, and three patients had three procedures. One patient had the second CA for the right seminal vesicle involvement only, and one patient had open pelvic lymph nodes dissection (PLND) in conjunction with CA. Additional therapy was given for 13 patients: 8 received HT, 3 received HT and/or radiation therapy, 1 received radiation therapy, chemotherapy, and ketoconazole, and 1 patient underwent robotic RP. Ethical approval from MD Anderson Cancer Center was obtained, and the study was carried out in compliance with the Helsinki Declaration. The patients were informed about the operation and consent was obtained. Biochemical recurrence after CA was defined as detectable measurement of PSA. Continence was defined as use of no pads or a small liner for security purposes only. Potency was defined as the ability to achieve an erection adequate for penetration with or without medication (i.e., phosphodiesterase-5 inhibitors, intracavernous injection, or vacuum). The median hospitalization and follow-up periods were 1 day (range 0–2) and 40.5 months (range 11–96).

**Table 1:** Demographic characteristics and oncological and functional outcomes of the study group

Variable	Value
Patients (*n*)	27
Age (years, median, range)	71 (48–80)
Initial PSA value (ng/ml, median, range)	6.2 (4–23.6)
Initial volume of prostate before CA (cc, median, range)	29 (15–53)
Volume of prostate after repeat CA (cc, median, range)	18 (10–48)
Gleason score at initial diagnosis	
6 (*n* %)	5 (19)
7 (*n* %)	13 (48)
8 or 9 (*n* %)	9 (33)
Initial clinical stage before first CA	
T1c (*n* %)	15 (55)
T2 (*n* %)	11 (41)
T3 (*n* %)	1 (4)
Initial therapy before first CA	
HT (*n* %)	6 (22)
HT and either RT or BT (*n* %)	5 (19)
RT (*n* %)	1 (4)
Interval between first CA and positive biopsy (median, months)	23 (6–63)
PSA value at positive biopsy after first CA (median, ng/ml)	2.7 (0.2–7.8)
Postoperative complications of repeat CA (*n* %)	
Continence (no pad usage)	24/26 (92)
Stricture	1/26 (3.84)
PSA value at last follow-up^a^	
Median value among patients with detectable PSA (ng/ml)	0.8 (0.2–50)
Patients with undetectable PSA (*n* %)	5/26^a^ (19)
Patients with PSA value <0.5 ng/ml (*n* %)	13/26 (50)
Patients with PSA value ≥0.5 ng/ml (*n* %)	8/26 (31 %)

*n* number of patients, *CA* cryoablation, *PSA* prostate-specific antigen, *HT* hormonal therapy, *RT* radiation therapy, *BT* brachytherapy

^a^One patient did not return for follow-up after the second CA

## Results

The procedure was well tolerated in all patients, and no intraoperative complications occurred. The PSA value before the first CA procedure was 5.6 ng/ml (range 0–15). Ten patients (37 %) had undetectable PSA after the first CA. Clinical variables assessed before and after each CA procedure are summarized in Table 2. One patient did not return for follow-up after the second CA. The median nadir PSA values after the first, second, and third CA were 0.65, 1.25, and 1.6 ng/ml, respectively. The median interval from first CA to first positive biopsy was 23 months (range 6–63), with median value of PSA 2.7 ng/ml (range 0.2–7.9). The median period between the first CA and the second CA was 27 months (range 7–78). The positive biopsy cores were located at the apex and/or base of the prostate in 65 % of patients undergoing the first CA, 70 % of patients undergoing the second CA, and 100 % of patients undergoing a third CA. Only one patient had failure of HT and chemotherapies and developed bone metastasis 14 months after the first CA. Five patients (5/26, 19 %) (two of them without additional therapy) had undetectable PSA level at the last follow-up, and 8 patients (8/26, 31 %) had detectable PSA values less than 0.5 ng/ml. Twenty patients (20/26.77 %) had PSA values less than 2 ng/ml at the last follow-up, and the median PSA value was 0.8 ng/ml (range 0.2–50) in all patients with detectable PSA.

**Table 2:** Pre- and postoperative clinical variables by cryoablation procedure

Variable	First CA	Second CA	Third CA
Patients (*n*)	27	27	3
Median PSA before CA (ng/ml, range)	5.6 (0–15)	3 (0–6.94)	2 (1.1–12.8)
Median total cores at biopsy before CA (*n*, range)	10 (4–14)	12 (6–92)	13 (12–60)
Median positive cores at biopsy before CA (*n*, range)	3 (1–12)	2 (1–9)	4 (1–7)
Median percentage of positive cores before CA (%, range)	33 (8–100)	10 (1–75)	8 (5–58)
Median cumulative length of cancer at biopsy before CA (mm)	16 (1–136)	4.5 (1–53.5)	17 (1–31)
Location of cancer at apex and/or base (*n* %)	15/23 (65)	19/27 (70)	3/3 (100)
Location of cancer at apex (*n* %)	6/23 (26)	12/27 (44)	1/3 (33)
Location of cancer at base (*n* %)	3/23 (13)	4/27 (15)	1/3 (33)
Undetectable PSA after CA (*n* %)	10/27 (37.03)	4/27 (14.81)	0
Nadir PSA after CA (ng/ml, range)	0.65 (0.1–4.9)	1.25 (0.2–7.9)	1.6 (0.4–4.5)
Duration of CA procedure (minutes)	115 (186–169)	100 (59–168)	130 (109–250)^a^

*n* number, *CA* cryoablation, *PSA* prostate-specific antigen

^a^One patient underwent open PLND with third CA

According to Clavien classification system, postoperative complications were seen in five patients (5/26, 19 %); three patients had grade I, one patient had grade II, and one patient had grade IIIb. One patient (4 %) developed a severe lower urinary tract symptom of acute retention, which started as irritative symptoms after the first CA and increased after the second and third CA; injury of the prostatic urethra was observed after the third CA. One patient (4 %) had urethral stricture with overactive bladder symptoms, and a transurethral bladder neck resection was performed. Three patients (11 %) developed mild perioperative complications—mild lower urinary tract symptoms, scrotal edema, and perineal abscess which resolved spontaneously. One patient developed neuropathy in the lower extremity and spinal stenosis due to radiation therapy that was given after the third CA. Two patients (8 %) had incontinence with the need for one pad per day (Table 1). Those two patients were primary cases without history of radiotherapy before CA. One of them had history of urethral stricture and underwent transurethral resection. Regarding erectile dysfunction, only 6 patients were potent before CA, and 21 patients either had no available data about erectile dysfunction or could not receive medication for it due to older age or comorbid diseases. However, 5 patients (19 %) could achieve erection with medication (3 with intracavernous injection, 1 with phosphodiesterase-5 (PDE-5) inhibitors, and 1 with a vacuum device) at the end of the second CA.

## Discussion

Albeit selection criteria for men undergoing prostate CA have yet to be definitely established, optimal candidates for this procedure generally include those with lower-stage, lower-volume disease, and PSA values less than 20 ng/ml [15]. Our findings demonstrate the validity of CA as an effective and safe treatment option not only for lower-risk patients but also for the intermediate- or even high-risk patients with localized prostate cancer. Similarly, Roberts et al. reported, in an analysis of a Medicare-linked database from 2004 to 2005, that 16.1 % of those who underwent CA therapy had high-risk disease [16]. In our series, only 5 patients (19 %) had a Gleason score <6 and 9 patients (33 %) had a Gleason score of either 8 or 9. The clinical stages for 5 patients (4 patients T2b, 1 patient T3) were also not confined to a low-risk group. One patient developed bone metastasis, 19 % of the patients had undetectable PSA level, and 50 % had a PSA level less than 0.5 ng/ml after CA. The patient who developed bone metastasis had a clinical stage of T2a, 100 % of cores positive, and a high volume (136 mm) of cancer at biopsy with a Gleason score of 8 and 5 years of neoadjuvant hormone therapy; thus, this patient had a poor prognosis and benefit from other kinds of therapies was unlikely. Three patients had progressive disease from T1c to T3 (seminal vesicle involvement) after CA; 1 patient underwent a second CA for the right seminal vesicles, and his last PSA test yielded an undetectable level, and the other patient, who had a history of radiation therapy before CA, underwent robotic RP without complication and at his last follow-up was under watchful waiting with a PSA level of 0.4 ng/ml. One patient had a positive lymph node after the second CA and underwent open pelvic lymph node dissection. A biopsy after the last CA detected no cancer, and his PSA value at last follow-up <0.1 ng/ml. An advantage of CA for intermediate- or even high-risk patients with localized prostate cancer is that it does not hinder patients from undergoing other kinds of salvage therapies such as robotic RP; Pisters et al. reported on the feasibility of RP after neoadjuvant CA [17]*.*

Cryotherapy can be used as a salvage treatment, and it is currently the only repeatable therapy for localized prostate cancer. Among salvage therapies, salvage RP and salvage CA represent the most feasible and effective therapeutic options for prostate cancer that recurs after radiation therapy. Salvage cryotherapy is performed four times as often as salvage RP, although both procedures remain underutilized for the management of recurrent prostate cancer [18, 19]. Pisters et al. performed a stratified control comparison of biochemical disease-free survival after salvage RP and salvage CA [20]. Compared to salvage CA, salvage RP resulted in superior biochemical disease-free survival, 21 % for salvage CA vs 61 % for salvage RP at 5 years [20]. Thus, salvage CA is best considered in older patients who decline to undergo salvage RP. However, there is no curative therapy in prostate cancer that is not responding to radiotherapy, and there is no definition of success for CA. The PSA level before salvage CA and Gleason score correlated with biochemical progression-free survival (bPFS): the initial PSA level of <0.6 ng/mL after salvage CA portends a favorable (67 % at 36 months) bPFS [21]. In our series, CA was used as salvage therapy for 12 patients (44 %, 6 of whom had previous radiation therapy (RT)) and as primary therapy for 15 patients. Although the previously reported intraoperative and postoperative complication rates have been high after salvage CA, our patients had no rectal injury, bowel bleeding, or hydronephrosis. Therefore, CA is not associated with the high intraoperative complication rate of other kinds of salvage therapies, and it is a feasible treatment modality in patients for whom initial therapies, including radiation and CA, have failed. Many recent studies have reported on the validity of salvage CA ablation after radiation therapy for prostate cancer [22]; however, we found only one study about the feasibility of repeat CA after the failure of the primary CA [23]. In one of these studies, de Castro and his colleagues compared salvage focal with standard salvage total CA for locally recurrent prostate cancer after primary radiation therapy [22]. They concluded that salvage focal and salvage total CA are feasible and safe with acceptable mid-term oncological outcomes and that morbidity was lower for salvage focal CA than for salvage total CA [22]. Focal therapy is a modification of the standard CA technique aiming to only treat the portion of the prostate gland which has the cancer with minimization of complications especially potency. This kind of therapy was introduced by Onik et al. [24] in 2004 and supported by many recent studies regarding its efficacy in the treatment of primary and radiorecurrent locally prostate cancer [25].

The postoperative complications occurring after CA have been widely studied in small single hospital-based studies. Urinary incontinence ranged from 1.3 to 9.5 % [9–11, 13, 26], urinary strictures from 1.7 to 17 % [10, 13], lower urinary tract obstruction and retention from 6.6 to 13 % [25], erectile dysfunction from 47 to 94.9 % [10, 11, 13], and fistula less than 1 % [10–13]. In our cohort of men receiving repeat CA, the majority of the postoperative complications were clinically insignificant. According to Clavien classification system, only one patient had grade IIIB complication and endoscopic management was done. No urinary or bowel fistula was observed. Incontinence occurred in two patients (8 %), with only one pad per day. One of them had had rectal abscess surgery 24 years before CA, and he suffered from mild nocturia after the first CA, which increased after the second CA. The second patient had urethral stricture with overactive bladder symptoms after the second CA; thus, bladder neck transurethral resection was done 32 months after the second CA. This patient had lower urinary tract symptoms before the first CA and had a history of urethral stricture after a fall from a tree; several urethral dilations had been done before the first CA. Thus, postoperative complications occurring after CA were not only attributable to the CA but also to the previous history. Another complication seen (one patient) was severe lower urinary tract symptoms and urinary retention after the third CA, due to injury of the prostatic urethra; thus, a Foley catheter was placed. This patient also had a history of irritative symptoms, which increased after the first CA and became severe after the third CA. Erectile dysfunction is a well-known side effect of CA; in our study, although the patients were older and the majority of the patients either were not potent before CA or were not interested in sex therapy, five patients could achieve erection with the help of medication after the last CA. The evaluation of erectile dysfunction in our study was suboptimal; therefore, it is difficult to make a definitive comment regarding potency. However, 19 % of the patients after repeat CA could achieve erections sufficient for penetration, which is a promising finding.

Location of cancer in the prostate base and/or apex is one of the greatest challenges for application of CA. The use of thermocouples in the apex and base to document adequate freezing temperatures could improve the results of salvage cryotherapy. In particular, the apex is sometimes difficult to visualize ultrasonographically and a thermocouple in both the right and left apical tissue may improve the effectiveness of salvage cryotherapy. In our study, the proportions of patients with cancer sited at the base and/or apex before the first, second, and third treatments were 65, 70, and 100 %, respectively (Table 2). Two patients who achieved undetectable PSA levels after the last CA had such cancers (one patient, cancer at the apex; and one patient, cancer at the base and apex). An undetectable PSA level is unlikely to be achieved after CA because a rim of PSA-producing periurethral tissue will invariably remain. Biopsy is the most definitive determination of failure or success; however, there is no consensus on when and at which PSA level should be performed.

## Conclusions

Our study demonstrates that repeat CA is both safe and effective. Cryotherapy as a primary or salvage option can be repeated with a modest complication rate and reasonable efficacy. Low- and high-risk localized prostate cancer can be treated with CA without hindering the patients from undergoing other kinds of salvage therapies. Base or/and apex of the prostate are the most common place for the recurrence after CA.
